# Population genetic structure of *Culex tritaeniorhynchus* in different types of climatic zones in China

**DOI:** 10.1186/s12864-024-10589-4

**Published:** 2024-07-05

**Authors:** Ye Zhang, Haifang Wang, Jun Du, Yandong Wang, Chuanhui Zang, Peng Cheng, Lijuan Liu, Chongxing Zhang, Ziwei Lou, Jingjing Lei, Jiahui Wu, Maoqing Gong, Hongmei Liu

**Affiliations:** 1 Shandong Institute of Parasitic Diseases, Shandong First Medical University & Shandong Academy of Medical Sciences, Jining, Shandong Province 272033 People’s Republic of China; 2 Zibo Center for Disease Control and Prevention, 255026 Shandong, People’s Republic of China

**Keywords:** *Culex tritaeniorhynchus*, Genetic diversity, *COI*, *Wolbachia*, Japanese encephalitis virus, Climate

## Abstract

**Background:**

*Culex tritaeniorhynchus* is widely distributed in China, from Hainan Island in the south to Heilongjiang in the north, covering tropical, subtropical, and temperate climate zones. *Culex tritaeniorhynchus* carries 19 types of arboviruses. It is the main vector of the Japanese encephalitis virus (JEV), posing a serious threat to human health. Understanding the effects of environmental factors on *Culex tritaeniorhynchus* can provide important insights into its population structure or isolation patterns, which is currently unclear.

**Results:**

In total, 138 *COI* haplotypes were detected in the 552 amplified sequences, and the haplotype diversity (*Hd*) value increased from temperate (0.534) to tropical (0.979) regions. The haplotype phylogeny analysis revealed that the haplotypes were divided into two high-support evolutionary branches. Temperate populations were predominantly distributed in evolutionary branch II, showing some genetic isolation from tropical/subtropical populations and less gene flow between groups. The neutral test results of HNQH (Qionghai) and HNHK(Haikou) populations were negative (*P* < 0.05), indicating many low-frequency mutations in the populations and that the populations might be in the process of expansion. Moreover, *Wolbachia* infection was detected only in SDJN (Jining) (2.24%), and all *Wolbachia* genotypes belonged to supergroup B. To understand the influence of environmental factors on mosquito-borne viruses, we examined the prevalence of *Culex tritaeniorhynchus* infection in three ecological environments in Shandong Province. We discovered that the incidence of JEV infection was notably greater in *Culex tritaeniorhynchus* from lotus ponds compared to those from irrigation canal regions. In this study, the overall JEV infection rate was 15.27 per 1000, suggesting the current risk of Japanese encephalitis outbreaks in Shandong Province.

**Conclusions:**

Tropical and subtropical populations of *Culex tritaeniorhynchus* showed higher genetic diversity and those climatic conditions provide great advantages for the establishment and expansion of *Culex tritaeniorhynchus.* There are differences in JEV infection rates in wild populations of *Culex tritaeniorhynchus* under different ecological conditions. Our results suggest a complex interplay of genetic differentiation, population structure, and environmental factors in shaping the dynamics of *Culex tritaeniorhynchus*. The low prevalence of *Wolbachia* in wild populations may reflect the recent presence of *Wolbachia* invasion in *Culex tritaeniorhynchus*.

**Supplementary Information:**

The online version contains supplementary material available at 10.1186/s12864-024-10589-4.

## Background

Climate change influences the pattern and extent of transmission of infectious diseases. Moreover, population mobility in the context of urbanization increases the risk of disease transmission in different regions. Meanwhile, high morbidity and mortality rates due to arbovirus infections have become a global health problem [1]. The incidence of endemic arboviral diseases caused by viruses such as West Nile virus (WNV), Dengue Fever Virus (DENV), Rift Valley Fever Virus (RVFV), and Zika Virus (ZIKV) has continued to rise in recent decades [2, 3]. *Culex tritaeniorhynchus Giles*, 1901, the primary vector of Japanese encephalitis virus (JEV), is widely distributed in Southeast Asia, the Middle East, Africa, and Europe [4]. It is estimated that more than 3 billion people worldwide are at risk of contracting JEV, with 67,900 cases of Japanese Encephalitis (JE) occurring annually, half of which occur in China [5]. Since 2008, with the inclusion of JE vaccine in China's Expanded Programme on Immunization (EPI), the incidence of JE has declined significantly, but sporadic and localized outbreaks are still frequently reported [6]. Cases of JE have been reported in all provinces of China except Qinghai [7]. Therefore, understanding the population genetic dynamics and genetic structure of mosquitoes is essential for assessing their ability to transmit diseases and develop effective vector surveillance tools.

Environmental and climatic changes (i.e., rainfall and temperature) have long been recognized as important promoters of mosquito-borne diseases [8, 9]. For instance, spatial changes like increased rice production in Asia and the construction of hydroelectric power plants create new breeding habitats for mosquitoes, thereby influencing mosquito population dynamics and elevating the risk of human exposure to vector populations [10]. It has been shown that habitat modification due to human activities promotes the biology of *Anopheles cruzii* and maintains the genetic diversity and structure of many terrestrial populations [11]. We have previously reported significant genetic differentiation between the *Culex pipiens pallen* populations in the hilly and mountainous of Shandong Province [12]. With the acceleration of urbanization in China, the mosquito originally adapted to the rural environment has gradually migrated to the urban environment, causing their population expansion or structure changes. At the same time, climate change affects patterns of seasonal and interannual variability. Global warming has expanded the habitat of *Culex tritaeniorhynchus*, gradually spreading from tropical/subtropical areas in southern China to temperate areas in the north and west [13]. Since the twenty-first century, data from the Chinese Center for Disease Control and Prevention (CDC) have shown that tropical/subtropical regions are the most severe areas of JE prevalence in China. The average incidence of JEV in Guizhou, Chongqing, Sichuan, and Yunnan ranked among the top four regions in China, while temperate regions such as Shaanxi and Henan also had high JE incidence, ranking fifth and sixth, respectively, showing a positive correlation with the widespread presence of *Culex tritaeniorhynchus* [14]. Given this information, China is facing an increasing public health threat from *Culex tritaeniorhynchus*. At present, molecular markers (e.g., isozymes of mitochondrial DNA, nuclear genes, microsatellite DNA, or mitochondrial genes) are widely used in mosquito species identification, population structure, molecular ecology, and phylogenetic relationships [15–17]. The mitochondrial cytochrome oxidase I (*COI*) gene, a popular marker gene for the study of species evolution, is considered the most valuable among the three subunits, with a stable gene structure, few deletions/insertions of gene fragments, and relatively conserved sequence composition. In addition, a recent study comparing the use of *COI* and the *18 s* gene explored results suggesting a higher success rate and greater potential for species identification with *COI* compared to ribosomal labeling [18].

It has long been recognized that natural *Wolbachia* infection is concentrated in *Culex pipiens pallens* and *Aedes albopictus* and has yet to be detected in *Culex tritaeniorhynchus* and *Aedes aegypti* [19]. However, recent studies have detected natural *Wolbachia* infections in *Aedes aegypti* in India, the United States, Malaysia and China. Natural *Wolbachia* infection was detected in *Culex tritaeniorhynchus* in Singapore and China [20–23]. *Wolbachia* is primarily transmitted vertically from mother to offspring and can induce cytoplasmic incompatibility (CI), orphan reproduction, male killing, and male feminization to manipulate host reproduction maximizing *Wolbachia* transmission [24]. *Wolbachia* inhibits viral replication in mosquitoes preventing infection from yellow fever, dengue, Zika, other arboviruses, and plasmodium [25]. The major strains of *Wolbachia* have different host specificities and symbiotic types, and 20 populations (A-T) have been identified by gene sequencing analysis [26]. However, only *Wolbachia* supergroups A and B were found in mosquitoes such as *Aedes albopictus*, *Armigeres subalbatus*, *Culex pipiens*, and *Culex tritaeniorhynchus* [23].

This study assessed the variation in the *COI* gene sequence in *Culex tritaeniorhynchus* in China. The key objectives of this study were (i) to construct phylogenetic relationships of *Culex tritaeniorhynchus* in different climatic type regions of China, and elucidate population genetic diversity, genetic differentiation, genealogical structure, and population dynamics characteristics. (ii) Evaluate the prevalence of symbiont *Wolbachia* in natural *Culex tritaeniorhynchus* populations. (iii) Assess the impact of environmental factors on the transmission of arboviruses.

## Materials and methods

### Adult mosquito collection and identification

The study was conducted in July and August of 2022 and 2023 in three climate type zones in mainland China (Table S1). The abbreviations of the 10 locations are as follows: HNTC, Tunchang; HNQH, Qionghai; YNXB, Xishuangbanna; HNHK, Haikou; GDGZ, Guangzhou; HNCS, Changsha; SDJN, Jining; SDZZ, Zaozhuang; SDZB, Zibo; ZGBJ, Beijing. The eastern, central, and southwestern regions of China are highly suitable habitats for *Culex tritaeniorhynchus* [14]. Shandong, Hunan, Yunnan, and Hainan used to be endemic areas for Japanese encephalitis [27]. Adult mosquitoes were collected using the Chinese Center for Disease Control and Prevention (CDC) mosquito trap light method in three different climatic type zones: tropical, subtropical, and temperate. Larvae collected were brought back to the laboratory and isolated and cultured according to the sampling point up to the adult stage. The species of field-collected adult mosquitoes were identified under a dissecting microscope according to established identification criteria [28]. All sorted-out mosquito specimens were washed with Phosphate buffer saline (PBS) solution and stored in 95% ethanol and stored at -80 °C.

### DNA extraction, *COI*/*WSP* gene amplification and sequencing

DNA from single mosquitoes was extracted using the Cador® Pathogen 96 QIAcube® HT instrument and a commercial kit following the manufacturer’s instructions (QIAGEN, Germany). Isolated DNA was stored at -80 ℃ and used as a template for gene amplification. *COI* was amplified using the LCO1490F (5′-GT CAA CAA ATC ATA AAG ATA TTG G-3′) and HCO2198R (5′-TAA ACT TCA GGG TGA CCA AAA ATC A-3′) primers [29]. PCR reactions were performed in 25 μL consisting of 12.5 μL of 2 × Phanta Max Master Mix (Vazyme Biotech Co., Ltd. China), 1.0 μL each of forward and reverse primers, 2 μL of DNA, and 8.5 μl of nuclease-free water. The PCR amplification reaction program was as follows: 94 ℃ for 3 min; 30 cycles of 94 ℃ for 3 s, 53 ℃ for 30 s, and 72 ℃ for 45 s; and finally, 72 ℃ for 5 min. PCR products were detected by electrophoresis using QIAxcel Connect (QIAGEN, Germany) and then verified by sequencing (Shanghai Biotech, China).

The *Wolbachia* surface protein gene (*WSP*) was amplified using the primers WSP81F (5′-TGG TCC AAT AAG TGA TGA AGA AAC-3′) and WSP691R (5′-AAA AAT TAA ACG CTA CTC CA-3′) to detect in vivo symbionts of *Wolbachia* in *Culex* [30]. The 25 μL PCR reaction contained 4 μL of DNA, 12.5 μL of 2 × Phanta Max Master Mix (Vazyme, Nanjing, China), 1 μL each of forward and reverse primers, and 6.5 μL of nuclease-free water. Thermal cycling consisted of a denaturation step at 95 ℃ for 2 min, followed by 35 cycles of 95 ℃ for 30 s, 55 ℃ for 45 s, and 72 ℃ for 30 s. The final extension was at 72 ℃ for 5 min. The amplification products were stored at 4 ℃, detected by QIAxcel Connect (QIAGEN, Germany) electrophoresis, and then sequenced by Sangyo Shanghai, China.

### Viral RNA extraction, amplification and sequencing

**Fig. 1:**
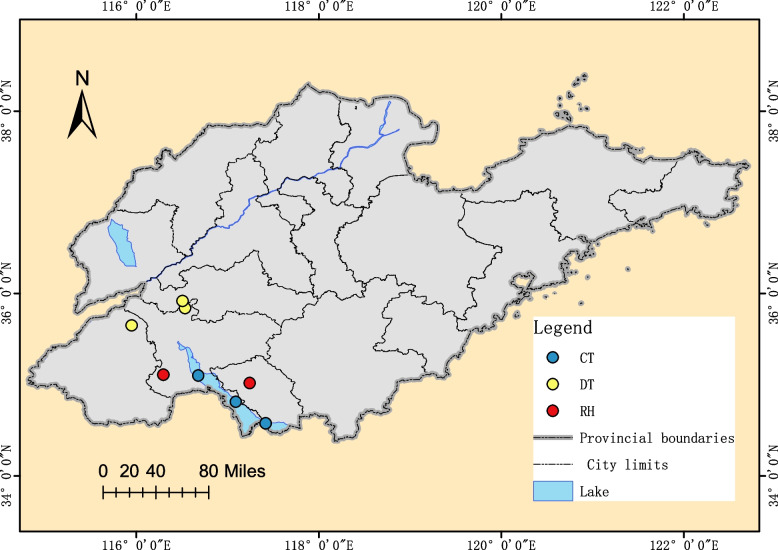
Sampling sites of *Culex tritaeniorhynchus* populations in different ecological environments in Shandong Province, China. CT Lotus Ponds, DT Paddy Fields, RH Irrigation canals

According to the "National Vector Pathogenesis Monitoring Program", we collected 1,156 *Culex tritaeniorhynchus* mosquitoes from three ecological environments (paddy fields, lotus ponds, and irrigation canals) in Shandong Province between June 2021 and August 2022 (Fig. 1). About 35 mosquitoes in each group were placed in 1.5 ml centrifugal tubes and completely ground with a grinder. This was used for the extraction of mosquito RNA using the Rneasy Mini Kit, following the manufacturer’s instructions (QIAGEN, Germany). Isolate RNA was preserved at -80 ℃. Mosquito and virus cDNA were prepared using the QuantiNovaTM Reverse Transcription Kit (QIAGEN, Germany). Heminested RT-PCR amplification of cDNA was performed using three universal primers targeting conserved regions designed by Xue et al. (Table 1) [31]. The first round of the PCR reaction system was 25 µl, including 12.5 μL 2 × Phanta Max Master Mix (Vazyme, Nanjing, China), 1 μL of XA-F1 (10 μmol/L), 1 μL of XA-R (10 μmol/L), 1 μL of cDNA and 9.5 μL of nuclease-free water. The PCR reaction conditions included a denaturation step at 95 ℃ for 3 min, followed by 30 cycles of 95 ℃ for 15 s, 53 ℃ for 15 s, and 72 ℃ for 30 s, with a final extension at 72 ℃ for 5 min. The second-round PCR reaction system was also 25 μL, including 12.5 μL 2 × Phanta Max Master Mix (Vazyme, Nanjing, China), 1 μL XA-F2 (10 μmol/L), 1 μL XA-R (10 μmol/L), 2 μL of the first-round PCR product and 8.5 μL nuclease-free water. The PCR reaction conditions included a denaturation step at 95 ℃ for 3 min, followed by 30 cycles of 95 ℃ for 15 s, 55 ℃ for 15 s, and 72 ℃ for 30 s, with a final extension at 72 ℃ for 5 min. PCR products were detected by electrophoresis using QIAxcel Connect (QIAGEN, Germany) and then verified by sequencing (Shanghai Biotech, China).

**Table 1:** Genetic diversity indices and neutrality tests based on *COI* gene of *Culex tritaeniorhynchus*

Region	*N*	*H*	*Hd*	*Pi*	*k*	*S*	Tajima's *D*	Fu's *Fs*	Fu & Li’s D* Test	Fu & Li’s F* Test
Tropical										
HNTC	50	16	0.934	0.00729	4.317	28	-1.023	-2.689	1.80799^*^	0.95967
HNQH	90	37	0.979	0.00994	5.887	56	-1.511^*^	-16.940^**^	2.22553^*^	0.76357
YNXB	22	7	0.848	0.03598	21.298	58	1.344	11.324	1.76481^*^	1.85053^*^
Mean	54	20	0.920	0.01774	10.501	47	-1.190	8.305	1.93278^*^	1.19126
Subtropical										
HNHK	58	22	0.915	0.00587	3.472	36	-1.832^*^	-9.834^**^	1.93067^*^	0.63252
GDGZ	20	4	0.674	0.00331	1.958	9	-0.785	1.725	1.37872^*^	0.87943
HNCS	56	22	0.958	0.01750	10.358	52	-0.290	-0.937	2.04575^*^	1.27757
Mean	45	16	0.849	0.00889	5.263	32	-0.969	-3.015	1.78505^*^	0.92984
Temperate										
SDJN	94	15	0.534	0.01541	9.121	45	0.117	4.466	2.12523^*^	1.54924
SDZZ	60	16	0.775	0.01852	10.965	40	0.924	2.962	1.97965^*^	1.80577^*^
SDZB	68	18	0.860	0.02632	15.582	39	2.980	5.241	2.00289	2.70478^*^
ZGBJ	34	11	0.898	0.02632	15.579	43	1.744	6.556	1.84364^*^	2.04914^*^
Mean	64	15	0.767	0.02164	12.812	42	1.441	4.806	1.98785	2.02723^*^

*N* Number of sequences, *H* Number of haplotypes, *Hd* Haplotype diversity, *Pi* nucleotide diversity, *k* average number of nucleotide differences, *S* number of polymorphic sites, *na* not applicable, *HNTC* Tunchang, *HNQH* Qionghai, *YNXB* Xishuangbanna, *HNHK* Haikou, *GDGZ* Guangzhou, *HNCS* Changsha, *SDJN* Jining, *SDZZ* Zaozhuang, *SDZB* Zibo, *ZGBJ* Beijing

^*^P < 0.05

^**^P < 0.01

### Data analysis

The obtained sequences were subjected to BLAST alignment with the NCBI sequence database to identify species and compared with voucher sequences and primers used in diagnostic PCR. Final species identification was based on high sequence identity (> 98%) with the voucher sequences in the database. Manual editing, if required, was performed using BioEdit v7.2.6. Low-quality sequences were excluded from data analysis and features were unweighted.

DnaSP v6 [32] was used to evaluate the number of haplotypes, haplotype diversity, nucleotide diversity, average number of nucleotide differences between sequences, number of polymorphisms, and insertions/deletions. Statistical neutrality tests were performed to detect DNA sequence variability if deviated from the expected evolutionary neutrality theory. Tajima's *D* test [33] is based on the difference between the estimated number of segregating sites and the mean pairwise difference; the D and F tests proposed by Fu and Li [34] are based on intraspecific molecular polymorphisms, and Fu's *FS* test is based on haplotype frequency distributions. Mismatch distributions were used to confirm any abrupt expansion in the *Culex* population. All calculations were performed in DnaSP v6. Arlequin 3.5.2 software [35], including the fixation index (*F*_*ST*_), gene flow (*Nm*), and AMOVA analyses of the populations. In this study, all haplotypes were analyzed using the PopART software, and a haplotype network diagram was constructed using the TCS algorithm. The unweighted pairwise group arithmetic mean (UPGMA) phylogenetic tree of COI sequences and neighbor-joining (NJ) phylogenetic tree of *WSP* sequences were constructed In MEGA11 [36].

Due to limited specimen collection from some areas, we downloaded *Culex tritaeniorhynchus COI* gene sequences from the GenBank-NCBI database [37] for parts of Hainan [38] and Hunan [39] Province (Table S1). We then constructed a preliminary haplotype network using the entire data set (PopArt).

Viral infections in mosquitoes were assigned by the maximum likelihood estimation (MLE) using the Excel add-in PooledInfRate v.4 program [40] and the rates are expressed as the number of infected mosquitoes per 1000 collected mosquitoes.

## Results

### Analysis of mtDNA genetic diversity

In total, 552 *COI* sequences were obtained from 276 *Culex tritaeniorhynchus* mosquitoes and used for genetic diversity and phylogenetic analyses. The PCR-amplified *COI* sequences were 592 bp long, with 110 variable sites. The average percentages of A, C, T, and G nucleotides were 29.8%, 16.1%, 38.6%, and 15.4%, respectively, and the content of A + T was higher than that of G + C, which was consistent with the characteristics of mitochondrial genes. A total of 138 haplotypes were defined, with a haplotype diversity (*Hd*) of 0.934 and a nucleotide diversity (*Pi*) of 0.02850, the average number of nucleotide differences was 16.870. Significant differences in haplotype indices were observed in *Culex tritaeniorhynchus* populations from different climatic regions (Table 1). Among them, the highest genetic diversity was found in *Culex tritaeniorhynchus* populations in the tropics, with mean values of 0.920, 0.01774, and 10.501 for *Hd*, *Pi*, and *k*, respectively.

### Haplotype network and phylogenetic analysis

**Fig. 2:**
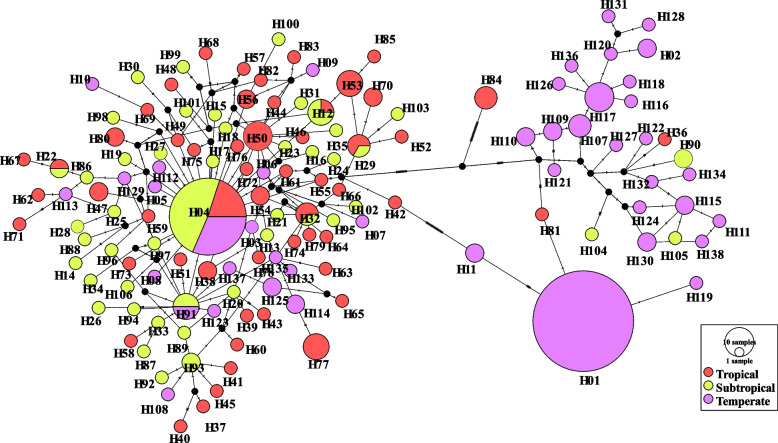
Phylogenetic network of 138 mitochondrial haplotypes of the *COI* gene in *Culex tritaeniorhynchus*. The haplotype network graph constructed based on Median-joining method. The size of each circle is proportional to its corresponding frequencies. Different colors indicate different populations. The number above the line referred to variable asynchronous numbers

**Fig. 3:**
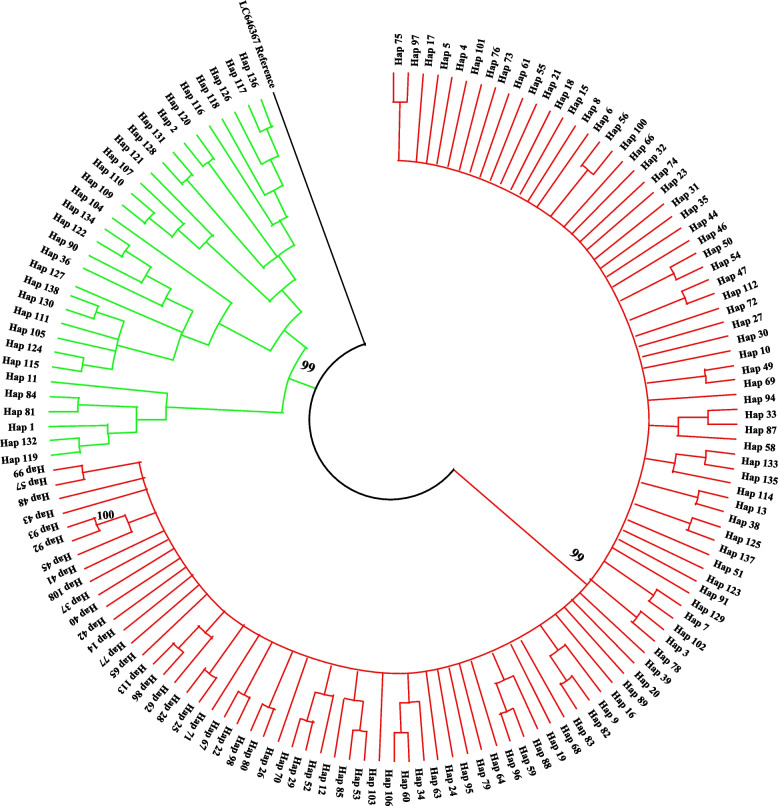
UPGMA dendrogram based on Nei’s unbiased genetic distance between the 138 haplotypes of *Culex tritaeniorhynchus*. The percentage of replicate trees in which the associated taxa clustered together in the bootstrap test (1000 replicates) are shown next to the branches. The tree is drawn to scale, with branch lengths in the same units as those of the evolutionary distances used to infer the phylogenetic tree

TCS network maps, which were constructed based on 552 *COI* sequences, showed clustered distribution patterns of 138 haplotypes in *Culex tritaeniorhynchus* populations (Fig. 2). Haplotypes in the first cluster H04 are the most common haplotype, distributed from tropical to temperate zones. The rest of the haplotypes are linked to the main haplotype by multiple base differences, with a high number of mutation sites between the derived and main haplotypes. This suggested high haplotype diversity in the *Culex tritaeniorhynchus* population. The majority of the other haplotypes (118/138;85.51%) are unique (Table 1). This may be due to limited sample selection or genetic variation in different climatic conditions. In addition, the unweighted pairwise group arithmetic mean (UPGMA) dendrogram based on unbiased genetic distances between haplotypes showed that the 138 haplotypes were divided into two clades with higher support (Fig. 3). The 107 haplotypes dominated by H04 form evolutionary branch I (red), which is mainly distributed in tropical/subtropical regions. While the other haplotypes formed evolutionary branch II (green), which is mainly distributed in temperate regions. This is consistent with the results of the median chain network.

### Genetic differentiation and population structure

**Fig. 4:**
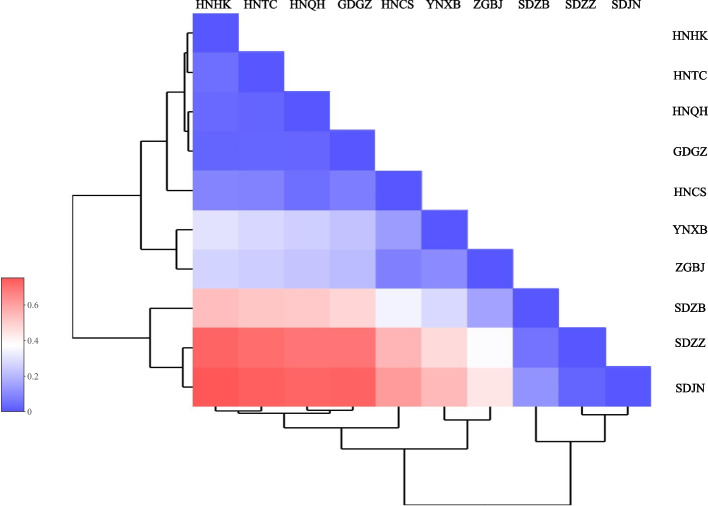
Heatmap of pairwise-*Fst* values for genotyping 10 *Culex tritaeniorhynchu*s populations in China

AMOVA analysis of the *COI* gene in the *Culex tritaeniorhynchus* population showed that 45.18% could be attributed to differences among populations; 9.23% to differences among populations within groups and 45.59% to differences within individuals (Table S2). The *F*_*ST*_ value can help assess the degree of genetic differentiation among geographic populations. The pairwise *F*_*ST*_ values between populations ranged from 0.02730 (SDJN, temperate region and SDZZ, temperate region) to 0.75214 (SDJN, temperate region and HNHK, tropical region). Of these, 25 pairs of populations were significantly genetically differentiated from each other (*F*_*ST*_ > 0.25; 55.55%), 4 pairs of populations were highly genetically differentiated from each other (0.15 < *F*_*ST*_ < 0.25; 8.88%), and 8 pairs were moderately genetically differentiated from each other (0.05 < *F*_*ST*_ < 0.15; 17.77%) (Table S3; Fig. 4). In addition, gene flow (*Nm*) estimates showed considerable variation between groups. The minimum gene flow (*Nm* = 0.16477) was found between HNHK and SDJN populations. In contrast, high gene flow (*Nm* = 17.81562) existed between SDJN and SDZZ populations. In summary, the highest level of genetic differentiation was observed between the HNHK and the SDJN populations.

### Historical population dynamics

Tajima's *D* and Fu's *Fs* neutrality tests have been found accurate in determining the historical dynamics of populations. Tajima's *D* and Fu's *Fs* values for both HNQH and HNHK populations were negative and significantly deviated from neutrality (Table 1), suggesting many low-frequency mutations in the populations and that the populations may be expanding. Additionally, Fu's *Fs* tests are particularly sensitive to demographic detection bias making it difficult to interpret whether the observed patterns are the result of positive selection or demographic history (e.g., population expansion). Population demographic expansion and spatial expansion were analyzed using mismatch distributions. Statistically significant squared deviation (SSD) values (0.07, *P* < 0.05) and raggedness index (Rag) (0.12, *P* < 0.05) were detected for the YNXB population. Mismatch distribution plots also showed the results as a single-peaked curve (Figure S1a-j), indicating historic rapid expansion in the YNXB population.

### Infection and genotyping of *Wolbachia*

**Fig. 5:**
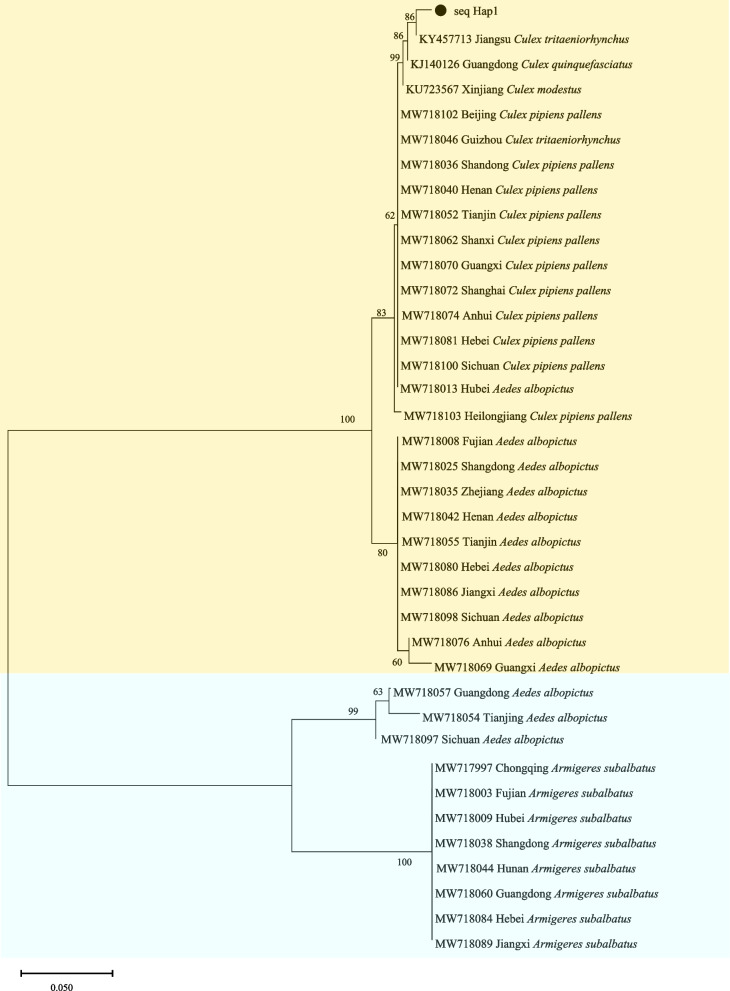
Neighbor-joining phylogenetic tree based on *Wolbachia WSP* gene partial obtained in this study and GenBank database. Strains identified in this study are identified with filled circles (●) for supergroup B. The numbers at the branches show bootstrap support (1000 replicates). The bar at the bottom of the figure denotes distance

A 540 bp fragment was amplified from 178 *Culex tritaeniorhynchus* samples using universal primers for *WSP*, confirming *Wolbachia* infection. We found that 2.24% (4/178) of the *Culex tritaeniorhynchus* samples were positive for *Wolbachia* infection. They were all also detected in the SDJN population (temperate region). A phylogenetic tree was constructed using the four sequences collected in this study and 37 reference sequences from the NCBI database (Fig. 5). The results showed that all four sequences from this study belonged to supergroup B and shared a recent common ancestor with the *Wolbachia* strain found in *Culex tritaeniorhynchus* in Jiangsu Province.

### Viral infection rate

Out of the 34 pools of *Culex tritaeniorhynchus* samples, 14 were positive pools. The sequences were aligned in the NCBI sequence database (using BLAST) and identified as JEV (Figure S2). The prevalence of JEV infection in CT (26.73/1000), DT (11.45/1000), and RH (8.90/1000) mosquitoes was calculated based on bias-corrected MLE (Table 2). The overall bias-corrected MLE infection rates of JEV in culicines were 15.27 per 1000 vectors, respectively.

**Table 2:** Bias-corrected maximum likelihood estimation (MLE) of JEV

Habitats	No. of individuals	No. of pools	No. of positive pools	Positive pool rate (%)	MLE per 1000 mosquitoes (95% CI)
CT	381	11	7	63.63	26.73 (12.06–57.16)
DT	402	12	4	33.33	11.45(3.80–28.35)
RH	373	11	3	27.27	8.90(2.40–24.28)
Total	1156	34	14	41.18	15.27(8.77–25.33)

*Abbreviations*: *CT* Lotus Ponds, *DT* Paddy Fields, *RH* Irrigation canals, *CI* Confidence interval

## Discussion

### High genetic diversity indicates continued expansion of tropical/subtropical populations

In this study, we examined the population structure of *Culex tritaeniorhynchus* in China based on mtDNA *COI* genes to assess potential genetic diversity arising from three climate zones. Haplotype diversity and nucleotide diversity are two important measures of species population diversity. The results indicate that populations in the tropics have higher genetic diversity. Li et al. also found 63 haplotypes and 70 variable loci with high haplotype diversity and nucleotide diversity (*Hd* = 0.98, *π* = 0.91) in *Culex tritaeniorhynchus* populations from tropical regions [38]. Similarly, a recent Turkish study based on the *COI* gene region detected 43 haplotypes with *Hd* and *Pi* values of 0,992 and 0,01433, respectively [41]. Of the 138 haplotypes detected, 65% (90/138) of the haplotypes were present only in the tropical/subtropical regions. High haplotype diversity in the tropics was also detected by Gao et al. [42] and Guo et al. [43] in *Aedes albopictus* populations in mainland China, and an environment more conducive to survival and continued dispersal may best explain this phenomenon. The haplotype network diagram shows that H04 is a dominant shared haplotype in three climatic zones, which indicates that H04 has gradually accumulated abundant ecological adaptive advantageous genes over a long period of evolution. On the other hand, the complex form of the haplotype network suggests a high rate of mutations in the genome, which helps insects acquire mutations favoring resistance faster in each generation, exacerbating the risk of insect-borne disease transmission [44]. In addition, several studies have found a strong correlation between the transmission of JEV and climate change, with temperature having multiple effects by affecting mosquito development and increasing virus replication in both hosts and vectors [13, 45, 46]. As the climate warms, the high genetic diversity of *Culex tritaeniorhynchus* promotes an increase in the geographic and seasonal distribution of mosquitoes, which in turn affects the risk of vector-borne virus transmission.

Southern China shares borders with Laos, Vietnam, and Myanmar, and frequent border trade and residential travel between these regions has led to the continued spread of *Culex tritaeniorhynchus*, enriching the diversity of local populations [47, 48]. This result was confirmed by a neutrality test, which found that Tajima's *D* and Fu's *Fs* for both HNQH and HNHK populations were negative and significantly deviated from neutrality, suggesting that there are many low-frequency mutations in the populations and that the populations may be expanding. Notably, the eggs of *Culex tritaeniorhynchus* cannot withstand dry conditions, so the warmer, wetter climates of the tropics/subtropics are more suitable for *Culex tritaeniorhynchus* than the drier air and lower temperatures of the temperate zones [4, 49]. Previous studies have shown that habitat adaptations among mosquitoes will increase to a large extent in the future with the expansion of river systems and land types. With climate change, *Culex tritaeniorhynchus* will gain a huge advantage in mosquito competition and changing conditions in the tropics/subtropics will favor its invasion [50]. Moreover, there is congruence between host mtDNA and the phylogeny of *Wolbachia* due to cytoplasmic hitchhiking driven by endosymbiont transmission [51]. Transmission of maternally inherited *Wolbachia* can lead to lower mitochondrial variation in populations. Previous studies have reported widespread *Wolbachia* infection in *Ae. albopictus* and *Culex pipiens*, and these populations had low mitochondrial haplotype diversity [52, 53]. Thus, we hypothesize that *Wolbachia* infection can also influence the high haplotype diversity of *Culex tritaeniorhynchus*.

### Population differentiation and genetic structure

Significant genetic differentiation was found between tropical, subtropical, and temperate populations, with less gene flow between populations. The AMOVA results showed that the proportion of genetic variation among individuals in the population (45.59%) and among the three regions (45.18%) was much higher than the proportion of genetic variation among groups within each region (9.23%). Xie et al. analyzed 21 *Culex tritaeniorhynchus* populations in mainland China, and within-population inheritance accounted for 98.8% of the total genetic variation [39]. China is a vast country with significant climatic differences, with the south being rainy, the north dry, the east humid, and the west arid. Therefore, the isolation effect of geographical distance and the ecological barriers formed by different climatic environments may inhibit gene exchange between mosquitoes in different regions. The UPGMA dendrogram-based results identified two genetic clusters of *Culex tritaeniorhynchus*, with temperate populations genetically distant from tropical/subtropical populations. Geographically scattered populations may lead to significant levels of mitochondrial genetic diversity for the *COI* gene in mosquitoes [54].

### *Wolbachia* infections in *Culex tritaeniorhynchus* mosquitoes

Notably, in this study, we detected *Wolbachia*-positive specimens in 178 *Culex tritaeniorhynchus* mosquitoes from three climatic zones in China. The overall prevalence of *Wolbachia* in *Culex tritaeniorhynchus* population was 2.24%, which was lower than in Guizhou, China (17.1%) [23]. Our detection of *Wolbachia* infection was done with a single female mosquito sample. However, previous studies reporting *Wolbachia* infection used undifferentiated male and female mosquito samples. Thus, this may partly explain the higher rates of *Wolbachia* infection reported in previous studies. Regardless, this study is the first to detect *Wolbachia* infection in Shandong Province. Dengue fever cases were often reported in Jinan and Qingdao in Shandong in 2017. To control the outbreak, large-scale environmental cleaning and pest control efforts were carried out across Shandong, with large quantities of insecticides being used to combat mosquitoes [55]. Liu et al. reported trends in insecticide resistance in *Culex* mosquitoes in Shandong Province over 20 years and found a dramatic increase in pyrethroid resistance [56]. *Wolbachia* infection in mosquitoes provides a new idea for mosquito control.

The results of the phylogenetic tree analysis revealed that *Culex tritaeniorhynchus* belongs to the *Wolbachia* B supergroup and shares a recent common ancestor with *Wolbachia* strains in *Culex tritaeniorhynchus* from Jiangsu, China. Vertical transmission is the primary mode of *Wolbachia* transmission between host generations. Alternatively, *Wolbachia* can be transmitted by host transfer (HS) between closely related and distantly related species. Gomes et al. reported an average similarity of 94.37% between *Tetranychus urticae* and *Ae. albopictus* in the *Wolbachia* B supergroup of *Wolbachia* direct homologs, but the similarity between host direct homologs was only 40.80% [57]. Ross et al. concluded that natural *Wolbachia* infection in *Ae. Aegypti* lacks sufficient evidence [58]. Therefore, the transmission route of *Wolbachia* in mosquitoes is not yet clear, requiring further studies.

### Environmental factors influence the spread of JEV

The emergence of mosquito-borne viruses with increased disease risk is largely influenced by various environmental factors. Among them, geo-environmental factors not only affect the genetic structure of mosquito populations, drug resistance, and carrying pathogens but also work in conjunction with climate change, leading to the exchange of pathogens between different hosts and regions, exacerbating the disease spread [12, 59]. Geography has a profound impact on the spread of mosquito-borne infectious diseases, which can be attributed to differences in geographic regions and changes in land-use patterns (e.g., urbanization, deforestation, agricultural development, tourist landscapes, etc.). A study conducted in Guangzhou, China, revealed that urbanization has expanded the habitat of *Ae. albopictus*. Furthermore, it was observed that urban areas exhibit faster larval and pupal development, along with higher larval and adult survival rates, in comparison to rural areas. More densely populated urban regions also experience elevated blood-feeding rates, thus enhancing their capacity to transmit arboviruses [60]. DeGroote et al. reported on WNV infections in Iowa, USA, spanning from 2002 to 2006, identifying 57 WNV-positive mosquito pools out of 3240 tested pools [61]. During the same time, they compared the distribution of human WNV cases in agricultural areas, wetlands, forests, commercial land, and residential land, finding that agricultural areas accounted for the highest percentage of cases. In this study, we found that the prevalence of JEV infection was significantly higher in *Culex tritaeniorhynchus* from lotus ponds than those from irrigation canal areas, highlighting the impact of land use. Sallam et al. examined the role of land cover types as suitable habitats for *Culex tritaeniorhynchus* using ecological niche modeling. They found that suitable habitats were closely associated with virus case dynamics [62]. Approximately 59.6 million people reside in 465,000 km2 of JEV-high-risk regions in China. Perhaps ecologically-based land-use planning, in combination with developments and sanitation improvements, could reduce both exposure to and populations of human commensal species, consequently limiting the spread of their pathogens.

In addition to changes in land use, other factors, such as the behavioral adaptability of vectors, contribute to the emergence and persistence of arboviruses. Most arboviruses have RNA as their genetic material, leading to a high mutation frequency that allows them to adapt to various vertebrate and invertebrate hosts [63, 64]. The lotus pond is located primarily in the Weishan Lake tourist area, which is historically one of the largest freshwater lakes in northern China and is connected to the Beijing-Hangzhou Grand Canal. The lake area is a tourist destination with high population mobility and a habitat for various birds. Such areas have become advantageous for the transmission of mosquito-borne diseases, completing the mosquito (virus)-bird-mosquito (virus) cycle of JEV transmission. In this study, the overall estimated JEV infection rate in *Culex tritaeniorhynchus* is 15.27/1000 mosquitoes, which is higher than the level reported in 2019 [65]. The risk of JE infection may have been reduced due to restrictions implemented during the novel coronavirus pneumonia outbreak. Nevertheless, our findings indicate a significant risk of a JE outbreak in Shandong Province currently. Additionally, in the study, all positive mosquito pools included both blood-sucking and unfed mosquitoes, and not all mosquitoes carrying JEV were infected. This suggests that the calculated prevalence rate might be overestimating the actual prevalence rate.

## Conclusions

In this study, we found that *Culex tritaeniorhynchus* populations in China are highly genetically diverse, particularly the populations in the tropicals. Through the phylogenetic analysis of haplotypes, two major haplotype clusters were observed, with small genetic differentiation and large gene flow within the same cluster, and large genetic differentiation and small gene flow among populations of different clusters. These findings indicate that *Culex tritaeniorhynchus* populations are influenced by distinct climatic regions. *Wolbachia* infection observed in *Culex tritaeniorhynchus* populations could offer insights for developing new vector control strategies. In addition, this study emphasizes that Shandong Province is at a high risk of a JEV outbreak. More attention should be paid to the impact of economic activities, agriculture, and geographic planning while designing control strategies for mosquito-borne virus diseases.

### Supplementary Information

Supplementary Material 1.

## Data Availability

All data supporting the findings of this study are included in this article. The newly generated sequences were submitted to the GenBank database under the accession numbers PP581486-PP581623.
