# Supplementary material for: Population genetic structure of Culex tritaeniorhynchus in different types of climatic zones in China
Source: BMC Genomics. 2024 Jul 5;25:673. doi: 10.1186/s12864-024-10589-4 (PMC11225206; doi:10.1186/s12864-024-10589-4)
Supplement: Supplementary file 1 — Supplementary Material 1. [file 12864_2024_10589_MOESM1_ESM.zip › supplementary material/supplementary material 4.docx]

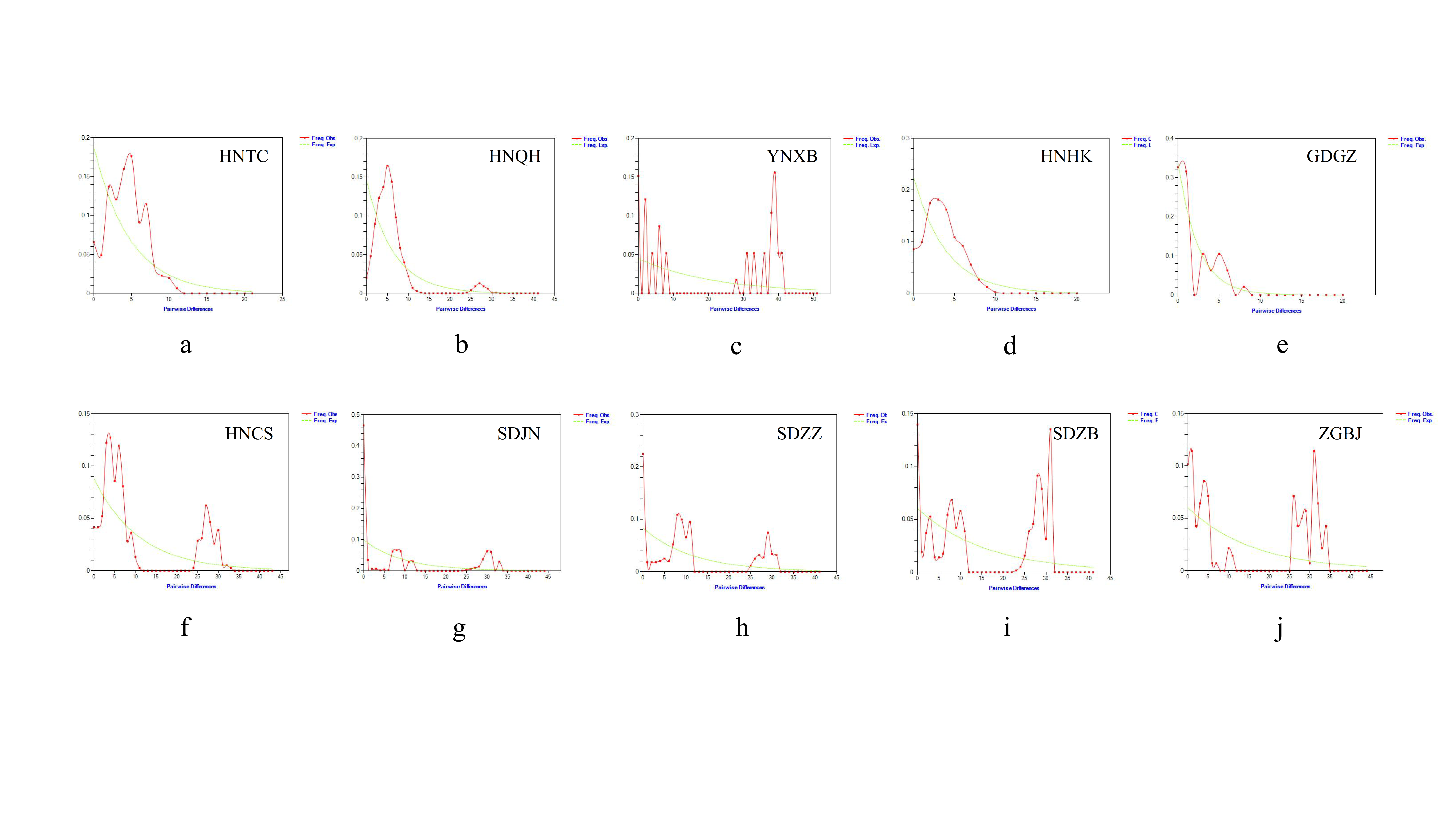


**Fig S1.** Mismatch distribution graphs for *Cx. tritaeniorhynchus* population based on *COI* sequences. The *x*- and *y*-axis show the number of pairwise differences and the frequency of the pairwise comparisons, respectively. The observed frequencies are represented by a dotted line. The solid line indicates the expected frequency under the assumption of a constant population model.


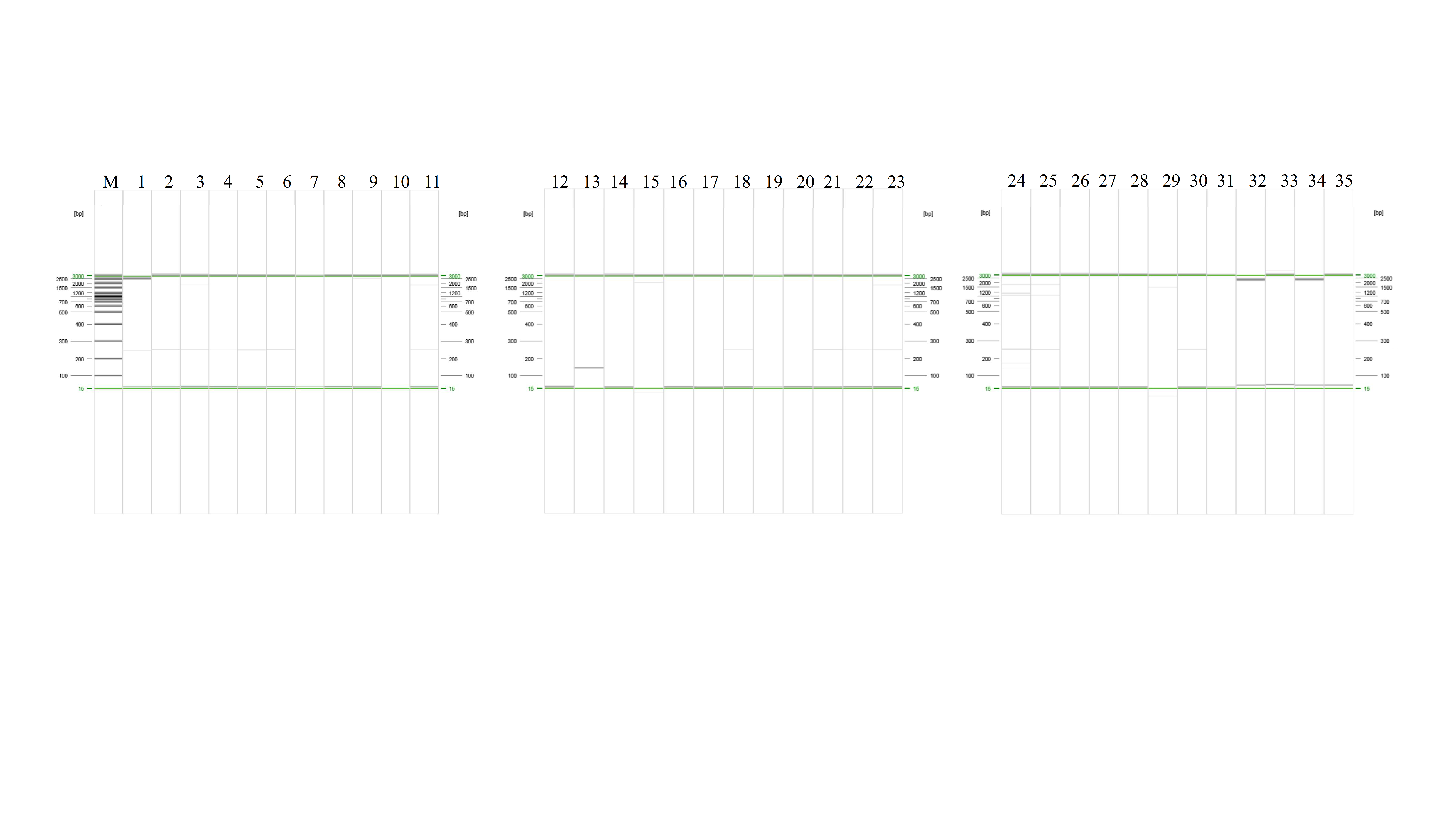


**Fig S2.** Detection of mosquito-borne flaviviruses by Heminested RT-PCR. Lanes 1–11, Lotus Ponds; Lanes 12–23, Paddy Fields; Lanes 24–34, Irrigation Canals; lane M, 2500bp size marker; Lane 35, blank.
